# Veterinary Societies

**Published:** 1883-01

**Authors:** 


### EDITORIAL DEPARTMENT.

### VETERINARY SOCIETIES.

IT is quite time that the veterinarians of this country unite to form a national organization which shall represent and look after the interests of the profession. At present there is a United States Veterinary Association which meets yearly and has some good members. It is officered and worked, however, chiefly by the faculty of a single veterinary college and in no sense represents the progressive and scientific element in the profession.

There are in this city and Brooklyn together only seventy-two registered graduates in veterinary science, yet we have at least four veterinary societies of various kinds, none of which do any creditable work. This state of affairs is the result of petty quarrels and jealousies which are disgraceful and injurious.

The veterinary profession is overrun with quacks. No body of men need so much organization for mutual protection, study and information. The hardest thing which a young veterinarian has to contend with is the cheap horse doctor, who goes around advertising his services and boasting of his skill.

If there were one society which all the educated veterinarians were interested in supporting much good might be done.

Veterinarians ought to remember that college quarrels and jealousies should not be allowed to interfere with professional work and progress.

